# *In vivo* adaptive antimicrobial resistance in *Klebsiella pneumoniae* during antibiotic therapy

**DOI:** 10.3389/fmicb.2023.1159912

**Published:** 2023-03-16

**Authors:** Shuangshuang Li, Xudong Feng, Min Li, Zhen Shen

**Affiliations:** ^1^Department of Laboratory Medicine, Ningbo Hospital, Ren Ji Hospital, Shanghai Jiao Tong University School of Medicine, Ningbo, China; ^2^State Key Laboratory for the Diagnosis and Treatment of Infectious Diseases, The First Affiliated Hospital, Zhejiang University School of Medicine, Hangzhou, China; ^3^Department of Laboratory Medicine, Ren Ji Hospital, Shanghai Jiao Tong University School of Medicine, Shanghai, China

**Keywords:** *Klebsiella pneumoniae*, antimicrobial resistance, adaptive evolution, *in vivo*, antibiotic therapy

## Abstract

*Klebsiella pneumoniae* is one of the leading pathogens contributing to antimicrobial resistance. The emergence of carbapenem-resistant *K. pneumoniae* (CRKP) has put the use of clinical antimicrobial agents in a dilemma. In particular, CRKP exhibiting resistance to ceftazidime/avibactam, tigecycline and colistin have raised great clinical concern, as these are the last-resort antibiotics for the treatment of CRKP infections. Within-host evolution is a survival strategy closely related to the emergence of antimicrobial resistance, while little attention has been paid to the *in vivo* genetic process of conversion from antibiotic-susceptible to resistant *K. pneumoniae*. Here we have a literature review regarding the *in vivo* evolution of resistance to carbapenems, ceftazidime/avibactam, tigecycline, and colistin in *K. pneumoniae* during antibacterial therapy, and summarized the detailed resistance mechanisms. In general, acquiring *bla*_KPC_ and *bla*_NDM_ harboring-plasmid, specific mutations in *bla*_KPC_, and porin genes, such as *ompK35* and *ompK36*, upregulation of *bla*_KPC_, contribute to the development of carbapenem and ceftazidime/avibactam resistance *in vivo*. Overexpression of efflux pumps, acquiring plasmid-carrying *tet (A)* variants, and ribosomal protein change can lead to the adaptive evolution of tigecycline resistance. Specific mutations in chromosomes result in the cationic substitution of the phosphate groups of lipid A, thus contributing to colistin resistance. The resistant plasmid might be acquired from the co-infecting or co-colonizing strains, and the internal environment and antibiotic selection pressure contribute to the emergence of resistant mutants. The internal environment within the human host could serve as an important source of resistant *K. pneumoniae* strains.

## Introduction

*Klebsiella pneumoniae* is a common pathogen for community-and hospital-acquired infections, such as pneumonia, urinary tract infection, and bacteremia (Podschun and Ullmann, 1998; Bengoechea and Sa Pessoa, 2019). The emergence of antimicrobial resistance in *K. pneumoniae* poses a serious threat to public health, particularly with the rise of carbapenem-resistant *K. pneumoniae* (CRKP). According to China Antimicrobial Surveillance Network, carbapenem resistance in *K. pneumoniae* increased rapidly from 3% in 2005 to more than 25% in 2019 (Hu et al., 2020). The irrational use of antibiotics in clinical practice has fostered the occurrence and spread of resistance to “old class antimicrobials.” Ceftazidime/avibactam, tigecycline, and colistin are now considered the last resort for the treatment of CRKP infections (Pournaras et al., 2016; Rabanal and Cajal, 2017; Shields et al., 2017b; Xu et al., 2022). However, the emergence of resistance to the last-resort antibiotics in CRKP has been repeatedly reported (Ah et al., 2014; van Duin et al., 2014; Shields et al., 2016, 2017a). More attention should be paid to the resistance of these types of antimicrobials. Besides, it is nerve-wracking to find that the antimicrobial resistance in *K. pneumoniae* is changing rapidly in response to *in vivo* microenvironmental stress during antimicrobial therapy (MacKenzie et al., 1997; Gaibani et al., 2022).

The prevailing view is that the growing prevalence of antimicrobial resistance is largely attributable to selection pressure from antibacterial drugs. However, our understanding of the *in vivo* development of antimicrobial resistance in *K. pneumoniae* is limited. While the majority of studies have focused on the epidemiology, risk factors, and treatment outcome of CRKP and other multi-drug resistant *K. pneumoniae* infections, relatively little attention has been paid to the *in vivo* genetic process underlying the conversion of a bacterial strain from antibiotic-susceptible to resistant. Within-host evolution is an important survival strategy, often associated with persistent or recurrent infections. Understanding the mechanisms that drive the *de novo* development of antimicrobial resistance in *K. pneumoniae* in patients during treatment is crucial for optimizing infection treatment and preventing the emergence of resistance. In this review, we focused on the antimicrobial drugs currently used in clinical practice, and summarized the adaptive evolution of antimicrobial resistance in *K. pneumoniae* under internal pressures. Our aim is to provide valuable insights into the emergence of antimicrobial resistance in *K. pneumoniae* during antibacterial therapy.

## *In vivo* adaptive resistance to carbapenem

### Acquisition of carbapenemase encoding genes

**Figure 1:**
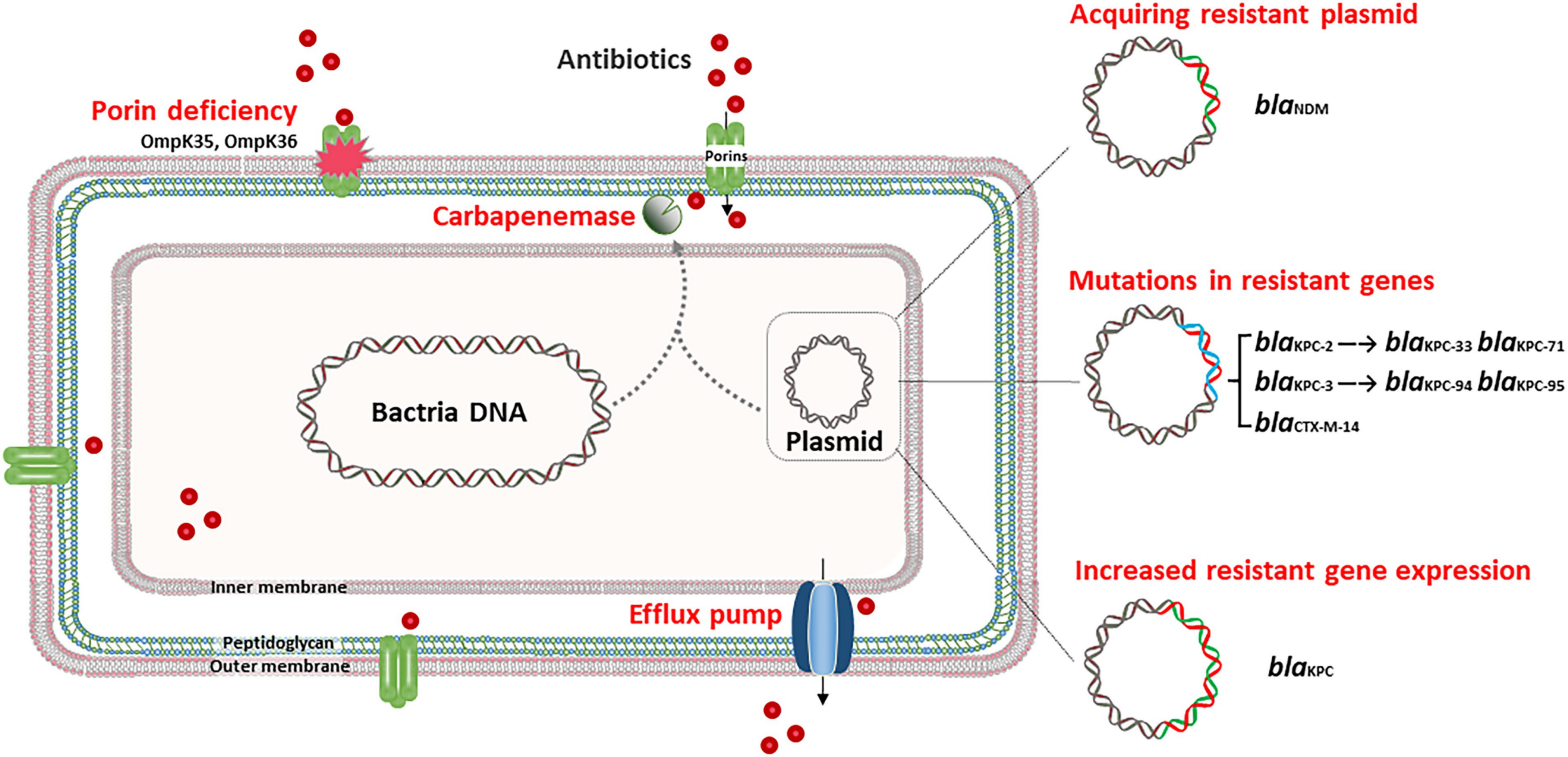
*In vivo* adaptive resistance to carbapenem and ceftazidime/avibactam. In general, acquiring *bla*_KPC_ harboring-plasmid, and specific mutations in porin genes, such as *ompK35* and *ompK36*, contribute to the development of carbapenem resistance *in vivo*. Acquiring *bla*_NDM_ harboring-plasmid, specific mutation in *bla*_KPC_ and upregulation of *bla*_KPC_ lead to *in vivo* ceftazidime/avibactam resistance.

The production of carbapenemase is the leading cause of carbapenem resistance in *K. pneumoniae*, with *K. pneumoniae carbapenemase* (KPC) being the most prevalent in several countries, including China (Hu et al., 2012; Giani et al., 2013; Pollett et al., 2014; Zhang et al., 2020). Ding et al. reported that the *in vivo* acquisition of *bla*_KPC-2_ led to carbapenem resistance in *K. pneumoniae* during antimicrobial therapy, and *bla*_KPC-2_ was acquired through horizontal transfer of an insertion sequence containing IS*Kpn6*-like, *bla*_KPC-2_ and IS*Kpn8* (Ding et al., 2016). Duplicative transposition might involve in the mobilization of this insertion sequence, since the transposase gene *tnpA* was located upstream of *bla*_KPC-2_ in KPC plasmids of the CRKP strains. Moreover, *in vivo* horizontal dissemination of the *bla*_KPC-2_ gene carried on IncL/M type conjugative plasmids has been observed among diverse Enterobacteriaceae clinical isolates with different genetic backgrounds, including *K. pneumoniae*, *E. coli*, and *E. cloacae complex* (Anchordoqui et al., 2015). These studies demonstrated that *in vivo* carbapenem resistance in *K. pneumoniae* can result from the horizontal transfer of a resistance plasmid or an insertion sequence (Figure 1).

Hypervirulent *K. pneumoniae* (hvKP) can also evolve into carbapenem-resistant hypervirulent *K. pneumoniae* (CR-hvKP) under long-term antibiotic treatment by acquiring a resistance plasmid. Recently, a report showed that a sequence type (ST) 218 hvKP strain developed into CR-hvKP in a patient by acquiring an IncFIIk *bla*_KPC_-harboring plasmid donated by a ST585 CRKP strain (Tang et al., 2021). *K. pneumoniae* ST218 is a single-locus variant of ST23 and belongs to clonal group 23, which comprises hypervirulent clones harboring the virulence factors of *iuc* locus, *iro* locus, and *rmpA*/*rmpA2* at high frequencies. The IncFIIk *bla*_KPC_-harboring plasmid is conjugative and carries five antibiotic resistance genes, *bla*_KPC-2_, *bla*_CTX-M-24_, *tet (A)*, *arr*-3, and *floR* (Table 1).

**Table 1:** Examples of studies on *in vivo* adaptive resistance in *Klebsiella pneumoniae.*

Resistant types	Initial strain types	Interval between S and NS isolates (days)	Sequence type (ST)	Underlying mechanisms	Region/Country
Carbapenem	KP	23	11	Acquisition of *bla*_KPC-2_-harboring Plasmid	Shanghai, China (Ding et al., 2016)
		7	11		
	KP	NA	37	Acquisition of *bla*_OXA-1_-harboring plasmid	Hunan, China (Li et al., 2017)
	hvKP	5	218	Acquisition of *bla*_KPC_-harboring plasmid	Hebei, China (Tang et al., 2021)
	KP	14	660	Porin deficiency	Zhejiang, China (Tian et al., 2020)
Ceftazidime/avibactam	KP	15/14	11	Mutation and reversion of *bla*_KPC_ genes	Beijing, China (Wang et al., 2021)
	CRKP	6	11	Mutations in *bla*_KPC_	Henan, China (Li D. et al., 2021)
	CRKP	10	101	Mutations in *bla*_KPC_	Italy (Gaibani et al., 2018)
	CRKP	21	NA	Mutations in *bla*_KPC_	Shanghai, China (Shen et al., 2022)
	MDR KP	23	383	Mutations in *bla*_CTX-M-14_	German (Both et al., 2017)
	CRKP	30	4496	*bla*_KPC-2_ duplication	Zhejiang, China (Han et al., 2021)
	CRKP	13–22	11	Increased gene expression of mutated *bla*_KPC_	Beijing, China (Sun et al., 2021)
	CRKP	11	11	Acquisition of *bla*_NDM-5_-harboring plasmid	Fujian, China (Huang et al., 2021)
Tigecycline	CRKP	41	11	Deletion of the *ramR* RBS	Shanghai, China (Ye M. et al., 2017)
	CRKP	340	1544	Upregulation of RamA and/or RarA	Taiwan, China (Lin et al., 2016)
		37	23		
		147	1526		
		7	660		
		5	45		
	hv-CRKP	50	11	Mutations of *ramR* and *lon*	Zhejiang, China (Jin et al., 2021)
	MDR KP	4	NA	*kpgABC* overexpression.	United States (Nielsen et al., 2014)
	CRKP	13	11	The *tet(A)* variant (S251A)	Zhejiang, China (Du et al., 2018)
	CRKP	NA	11	Plasmid harbors the *bla*_KPC-2_ and *tet(A)* variant genes	Zhejiang, China (Zhang et al., 2018)
	CRKP	29	11	The *rpsJ* variant (V57L)	Zhejiang, China (He et al., 2018)
Colistin	CRKP	30	258	Inactivation or deletion of the *mgrB* gene	Italy (Cannatelli et al., 2013)
	CRKP	12	512	Mutations of *pmrB*	Italy (Cannatelli et al., 2014)
	hv-CRKP	50	11	Mutations of *pmrB*, *phoQ*, and *mgrB* genes	Zhejiang, China (Jin et al., 2021)

KP, Klebsiella pneumoniae; CRKP, carbapenem resistant Klebsiella pneumoniae; hvKP, hypervirulent Klebsiella pneumoniae; ST, sequence type; S, susceptible; NS, not susceptible; RBS, ribosomal binding site; MDR, multi-drug resistance; BSI, bloodstream infection; NA, not available.

### Porin deficiency

Outer membrane protein (OMP) is the main component of the outer membrane of gram-negative bacteria, including porin and lipoprotein. Porins typically aggregate to form pores, enabling the passage of small hydrophilic molecules, such as β-lactams, across the membrane (Pagès et al., 2008). The porins of *K. pneumoniae* mainly include OmpK35 and OmpK36 (Hernández-Allés et al., 1999). The deficiency of OmpK35, known as OmpF, is reported widely reported in Enterobacteriaceae strains (Gravey et al., 2020). While OmpK35 is not thought to be the primary pathway for *K. pneumoniae* resistance (Shields et al., 2015; Pagès et al., 2016). The function of OmpK35 should be further studied *in vivo* circumstance of *K. pneumoniae*. OmpK36 is classified as a member of the OmpC porin group, and functions as a non-specific, passive diffusion pore (Ye Y. et al., 2017). The loss of OmpK36, coupled with extended-spectrum β-lactamases (ESBLs) and/or AmpC production, can result in carbapenem resistance in *K. pneumoniae* (Hamzaoui et al., 2018). Tian et al. (2020) reported three *K. pneumoniae* strains successively isolated from one patient during hospitalization and found that the final strain developed carbapenem resistance after 14-day of imipenem treatment. This particular CRKP strain exhibited OmpK36 deficiency due to a premature stop codon in the *ompK36* gene. This study highlights that the alteration of outer membrane porins due to the 14-day use of imipenem plays a potential role in leading to clinical presentation of carbapenem resistance (Figure 1). In addition, some researchers have gradually discovered that porins are also associated with ceftazidime/avibactam resistance of *K. pneumoniae* (Xu et al., 2022), which reflects the important role of porins deficiency in the adaptive evolution of *K. pneumoniae.*

## *In vivo* adaptive resistance to ceftazidime/avibactam

Ceftazidime/avibactam is a novel β-lactam/β-lactamase inhibitor combination, which has been approved for the treatment of complicated intra-abdominal infections and urinary tract infections in 2015 (Zasowski et al., 2015; Shirley, 2018). Avibactam exhibits activity against Ambler class A enzymes (including ESBLs and KPC) and some Ambler class C and D (e.g., OXA-48) enzymes, but it is ineffective against class B enzymes like New Delhi metallo-β-lactamase (NDM) (Shirley, 2018). Though ceftazidime/avibactam displayed potent activity against KPC-producing *K. pneumoniae* (KPC-KP), the emergence of ceftazidime/avibactam resistance in clinical strains during antimicrobial treatment has been repeatedly reported (Humphries et al., 2015; Shields et al., 2016; Both et al., 2017).

### Specific mutations of *bla*_KPC_

The major *in vivo* adaptive ceftazidime/avibactam resistance mechanism is the emergence of specific mutations in *bla*_KPC_ (Figure 1). A report from China revealed that following a 6-day course of ceftazidime/avibactam treatment, a ST11 *K. pneumoniae* strain developed ceftazidime/avibactam resistance, owing to the mutation of KPC-2 to KPC-33, with a substitution of D179Y within the Ω loop (Li D. et al., 2021). The Ω loop is located at residues 164–179, which is an essential domain for class A β-lactamases, and the substitution of D179Y may be detrimental to the binding of avibactam (Gaibani et al., 2021; Wang et al., 2021). Another study conducted in Italy showed that *in vivo* development of ceftazidime/avibactam resistance in *K. pneumoniae* was also linked to the D179Y substitution in KPC-3, which emerged after 17-day of ceftazidime/avibactam treatment (Gaibani et al., 2018). Similarly, *in vivo* development of KPC-71, KPC-76, KPC-94, and KPC-95-mediated ceftazidime/avibactam resistance during antimicrobial treatment has also been reported in ST11 and ST512 KPC-KP (Li X. et al., 2021; Guzmán-Puche et al., 2022; Shen et al., 2022). Of note, the low antibiotic pressure may have selected hybrid subpopulations of KPC-KP due to the high adaptability of KPC to ceftazidime/avibactam. For example, a study reported that KPC-KP strains isolated from bronchoalveolar lavage harbor *bla*_KPC-3_ and T243M mutations, while those isolated from the blood have D179Y mutation (Gaibani et al., 2021). Except for KPC, the *in vivo* emerging P170S exchange in CTX-M-14 has also been associated with elevated ceftazidime/avibactam MICs for independent *K. pneumoniae* isolates, but this substitution was not sufficient for full resistance (Both et al., 2017).

Interestingly, KPC mutations mediating ceftazidime/avibactam resistance are generally associated with the restoration of carbapenem susceptibility (Haidar et al., 2017; Shields et al., 2017a), and this kind of reversion may be dynamic. As illustrated in a recent study, the infection began with a KPC-2-producing *K. pneumoniae*. After treatment with ceftazidime/avibactam, the strain switched to a KPC-33 mutant (D179Y), which restored carbapenem susceptibility. However, the restored carbapenem susceptibility *in vivo* was not stable and the subsequent use of imipenem against KPC-33-producing *K. pneumoniae* infection resulted in a reversion of KPC-2 producers (Wang et al., 2021). The selective pressure of antibiotics in the mutation and reversion of *bla*_KPC_ genes may lead to the dynamic change of KPC enzymes and the emergence of resistance to ceftazidime/avibactam and carbapenems.

### Increased *bla*_KPC_ gene expression

Increased gene expression and copy number of *bla*_KPC_ can lead to ceftazidime/avibactam resistance in *K. pneumoniae*. A study reported that a novel ST4496 strain, which is a novel ST closely related to ST11, displayed ceftazidime/avibactam resistance after 1 month of ceftazidime/avibactam treatment. Sequencing analysis showed that there was duplication of *bla*_KPC-2_ on a 108 kb IncFII KPC plasmid due to unequal crossover of the IS26 composite transposon, resulting in elevated levels of *bla*_KPC-2_ expression (Han et al., 2021). The increased expression of KPC carbapenemase could lead to enhanced hydrolysis of ceftazidime, as avibactam may not be able to completely inhibit the higher amounts of KPC. Besides, the increased gene expression of *bla*_KPC-51_ also leads to high-level ceftazidime/avibactam resistance (Sun et al., 2021).

### Acquiring *bla*_NDM-5_-harboring plasmid

Acquiring *bla*_NDM-5_-harboring plasmid could also lead to ceftazidime/avibactam resistance, since avibactam could not inhibit NDM. One study observed that a ST11 CRKP strain displayed ceftazidime/avibactam resistance after 11-day of treatment. This strain harbored *bla*_KPC-2_, *bla*_SHV-182,_ and *bla*_TEM-1B_, and it acquired an additional IncX3 *bla*_NDM-5_-harboring plasmid when compared with the corresponding susceptible isolate. This *bla*_NDM-5_ plasmid is conjugative and could be successfully transferred into *E. coli* J53 with high frequency (Huang et al., 2021).

## *In vivo* evolution of tigecycline resistance

**Figure 2:**
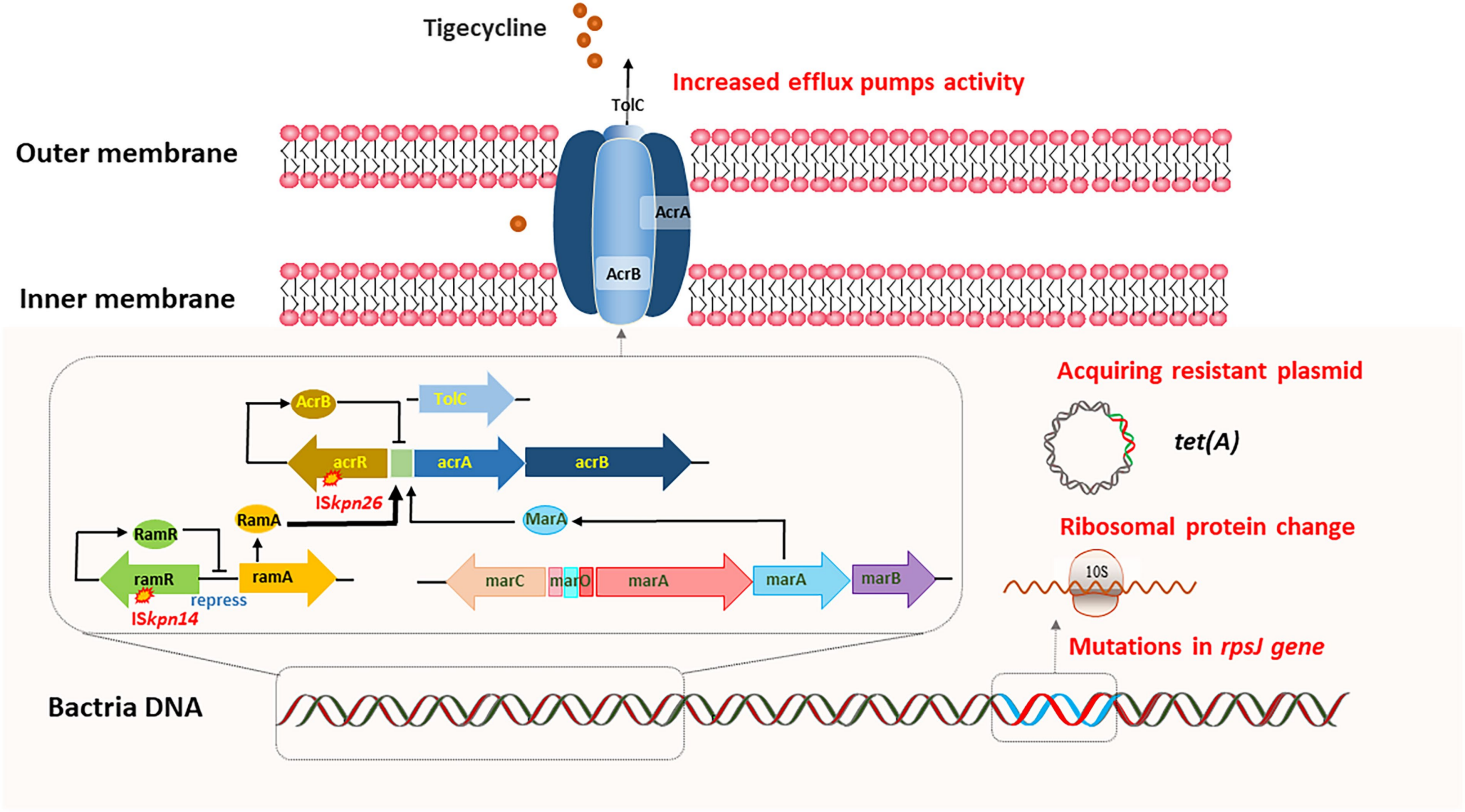
*In vivo* evolution of tigecycline resistance. Overexpression of efflux pumps, acquiring plasmid-carrying *tet(A)* variants, and ribosomal protein change can lead to the adaptive evolution of tigecycline resistance.

Tigecycline is a derivative of the semi-synthetic tetracycline minocycline, which was approved in China in 2012 (He et al., 2015; Alhashem et al., 2017). It has a broad antibacterial spectrum and displays potent antibacterial activity against gram-positive cocci, gram-negative bacilli (excluding *Pseudomonas aeruginosa* and some *Proteus*), anaerobic bacteria, and atypical pathogens (Society of Clinical Microbiology and Infection of China International Exchange and Promotion Association for Medical and Healthcare, Clinical Microbiology Group of the Laboratory Medicine Society of the Chinese Medical Association, Clinical Microbiology G.I.S. ofthe C. M, 2020). Tigecycline mainly binds to the bacterial ribosome 30S subunit, preventing aminoacyl-tRNA from entering the ribosome A site, thus exerting an antibacterial effect by inhibiting bacterial protein synthesis (Velkov et al., 2010). Recently, tigecycline-resistant *K. pneumoniae* strains have been frequently reported (Sun et al., 2013). Overall, the most commonly reported mechanisms of tigecycline resistance include overexpression of efflux pumps, acquisition of plasmid-carrying *tet (A)* variants and ribosomal protein change (Figure 2).

### Increased efflux pumps activity

Efflux pumps can actively squeeze drugs out of cells, and resistance nodulation-cell division (RND) type efflux pump, including AcrAB-TolC and OqxAB, is the dominant drug-associated efflux pump in gram-negative bacteria (Nikaido, 2018). AcrAB-TolC is predominantly related to tigecycline resistance in CRKP (Masi et al., 2019). AcrAB-TolC is mainly composed of three parts: membrane fusion protein (AcrA), efflux transporter (AcrB), and outer membrane channel protein (TolC). Meanwhile, the expression of the AcrAB-TolC is regulated by a variety of regulatory factors, including *acrR*, *ramA*, and *marA* (Wang et al., 2001; Keeney et al., 2007). Studies have shown that bacteria can sense flux rate and regulate efflux pumps to survive the environmental antibiotic challenge, suggesting that resistance mechanisms *in vivo* may differ from those *in vitro* (Fritz et al., 2015). Understanding the mechanisms underlying the *de novo* development of tigecycline resistance in patients is challenging. One strategy is to perform a longitudinal study by isolating *K. pneumoniae* strains from patients before, during and after tigecycline therapy. Ye et al. reported that the deletion of *ramR* ribosomal binding site (RBS) could lead to *in vivo* development of tigecycline resistance in *K. pneumoniae* after more than 40 days of tigecycline therapy (Ye M. et al., 2017, 2022). RamR exerted negative regulation on *acrAB* gene expression, and this 12-bp deletion abolished RamR protein production, resulting in high levels of *acrAB* expression and tigecycline resistance.

In another study, researchers identified five paired clinical isolates of *K. pneumoniae* that were initially tigecycline-susceptible, but later developed into tigecycline-non-susceptible (Lin et al., 2016). They found that tigecycline-non-susceptibility was associated with upregulation of RamA and/or RarA and the corresponding AcrAB-TolC and/or OqxAB efflux pump (s), respectively. Furthermore, various mutations in *ramR* and *oqxR* lead to *ramA* and *rarA* overexpression. Meanwhile, AcrAB-TolC efflux pump-mediated tigecycline resistance has also been confirmed in CR-hvKP strains. ST11-KL64 CR-hvKP developed tigecycline resistance due to the mutation of *ramR* during tigecycline therapy, and a novel frameshift mutation of *lon* was identified in the high-level tigecycline-resistant strain (Jin et al., 2021). In addition, Nielsen et al. also reported a new efflux pump operon, *kpgABC*. An insertion sequence (IS5) was correlated with an elevated *kpgABC* expression, which led to the *in vivo* development of tigecycline nonsusceptibility in *K. pneumoniae* (Nielsen et al., 2014).

### Acquiring plasmid-carrying *tet (A)* variants

The widely disseminated plasmid-carrying *tet (A)* variants in *K. pneumoniae* have greatly contributed to tigecycline resistance (Chiu et al., 2017). Tet (A) is one of the most common major facilitator superfamilies (MFS) efflux pumps. Tigecycline therapy could upregulate the expression of *tet (A)* in tigecycline-susceptible CRKP, leading to the development of tigecycline resistance. Du et al. (2018) reported tigecycline-resistant ST11 CRKP isolates from a 56-year-old female patient during tigecycline therapy. One amino acid substitution S251A in TetA was identified in the tigecycline-resistant isolates. Subsequent transformation experiments confirmed the contribution of this TetA variant (S251A) to tigecycline resistance and the *tetA* gene was located on a transferable plasmid. Another study reported that clinical CRKP strains carrying a conjugative plasmid harboring the *bla*_KPC-2_ and *tet (A)* variant genes readily evolved into tigecycline-resistant CRKP upon treatment and persisted in the human gastrointestinal tract (Zhang et al., 2018).

### Ribosomal protein change

Mutations in *rpsJ* encoding ribosomal protein S10, the target site of tigecycline, have also been associated with tigecycline resistance (Villa et al., 2014). He et al. (2018) monitored a 59-year-old male patient infected with ST11 KPC-producing *K. pneumoniae*. They identified the V57L amino acid substitution in *rpsJ*, and confirmed that this mutation was the main cause of tigecycline resistance through transformational complementation assay. This study demonstrated that the evolution of the *rpsJ* gene could lead to tigecycline resistance in CRKP during tigecycline therapy. Since this gene is located on the chromosome, it provides a clinical warning that under the selective pressure of tigecycline, *rpsJ* mutations may occur, resulting in drug resistance and treatment failure.

## *In vivo* evolution of colistin resistance

Polymyxins (polymyxin B and colistin) are the key drugs for the treatment of infections caused by CRKP (Livermore et al., 2011; Petrosillo et al., 2013). However, the emergence of colistin-resistant *K. pneumoniae* has been increasingly reported (Giani et al., 2013; Ah et al., 2014). There are some reports of *K. pneumoniae* acquiring colistin resistance under *in vivo* selective pressure.

**Figure 3:**
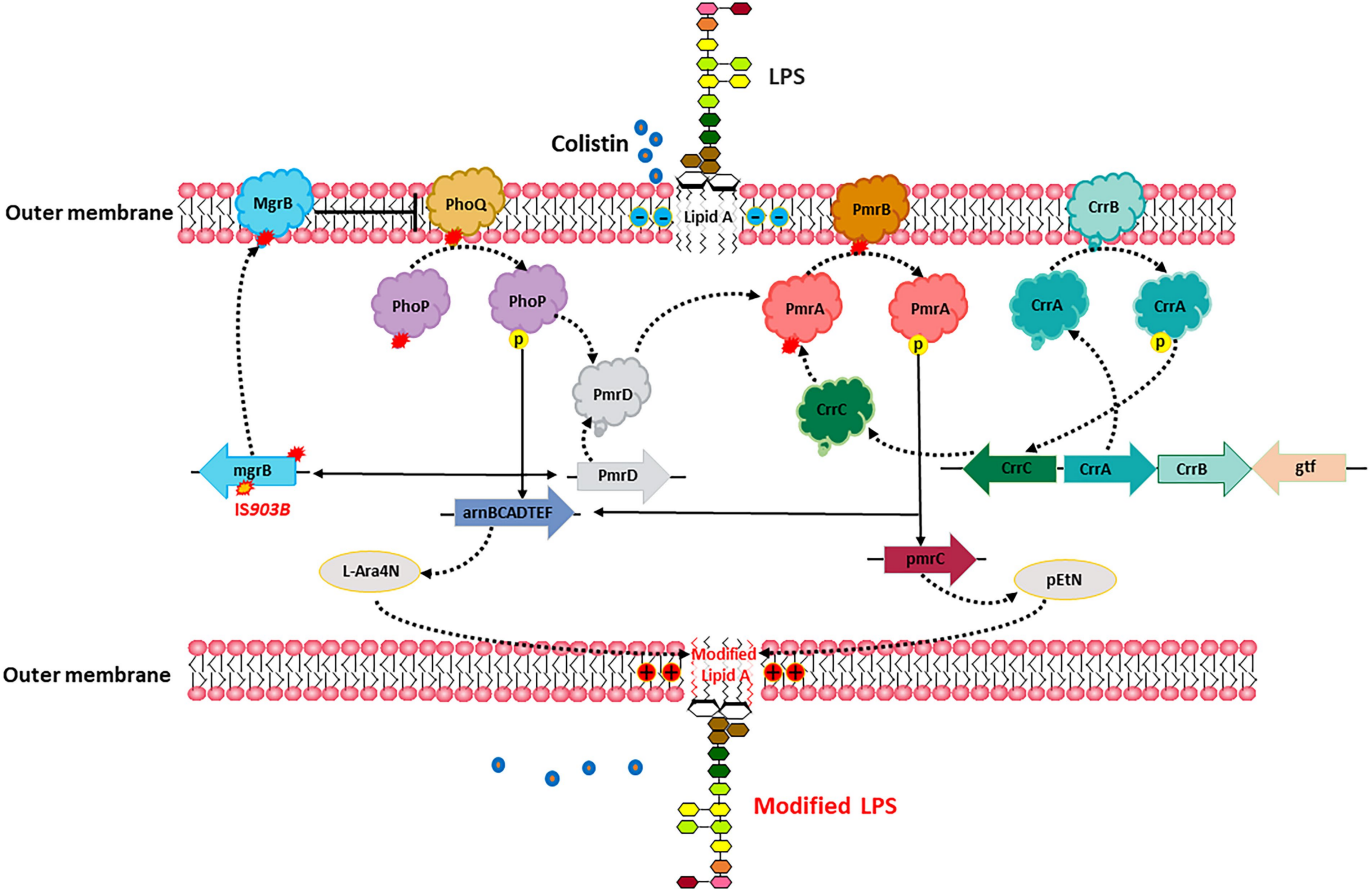
*In vivo* evolution of colistin resistance. Specific mutations in chromosomes led to the cationic substitution of the phosphate groups of lipid A, contributing to colistin resistance.

The outer membrane of gram-negative bacteria is the action site of polymyxins due to its affinity for the phosphate group of lipid A. Lipid A is the hydrophobic part located in the outer monolayer of the outer membrane and is synthesized by a series of enzymes encoded by the *lpx* gene cluster. Lipid A has a negative charge due to the presence of free phosphate groups, while polymyxin has a high affinity for the negative charge of lipid A (Li et al., 2019). As shown in Figure 3, two ionic groups, pEtN and L-Ara4N, can impact the affinity between polymyxin and the outer membrane and lead to resistance. The expression of these two ionic groups is controlled by LPS-modifying enzyme genes *pmrC*, *pmrE*, and *pmrHFIJKLM*, which is then controlled by PmrAB and PhoPQ two-component system (Kox, 2000; Lee et al., 2004; Olaitan et al., 2014). In addition, CrrAB and MgrB are also involved in this regulatory axis. CrrAB could regulate the expression of PmrAB and PhoPQ (Olaitan et al., 2014), while MgrB is a negative regulator of the PhoPQ signaling system. The mutations in certain genes are involved in the regulation of these signaling pathways, such as *pmrAB*, *phoPQ*, *mgrB*, *crrB*, which can lead to colistin resistance (Cheng et al., 2010; Cannatelli et al., 2013; Chen and Groisman, 2013; Xie et al., 2022).

Comparative genomic analysis of a pair of sequential KPC-KP isolates from the same patient, including a colistin-susceptible isolate and a colistin-resistant isolate selected after colistin exposure, revealed that insertional inactivation of the *mgrB* gene is a genetic mechanism for acquired colistin resistance (Cannatelli et al., 2013). Another study found a non-synonymous nucleotide substitution in the *pmrB* gene that resulted in a leucine-to-arginine substitution at amino acid position 82 in CRKP-infected patients treated with low-dose colistin (Cannatelli et al., 2014). This substation upregulated transcription of *pmrA* and *pmrK*, which is part of the *pmrHFIJKLM* operon responsible for modification of the colistin lipopolysaccharide target. Besides, the mutations of *pmrB*, *phoQ* and *mgrB* genes also account for the in-host evolution of CR-hvKP to colistin resistance (Jin et al., 2021).

## Conclusion

Our work summarized *in vivo* adaptive evolution of antimicrobial resistance in *K. pneumoniae* during antimicrobial therapy in currently clinical practice. We further described the underlying mechanisms of evolved resistance to carbapenems, ceftazidime/avibactam, tigecycline, and colistin within human hosts. In general, acquiring *bla*_KPC_ and *bla*_NDM_ harboring-plasmid, specific mutations in *bla*_KPC_, and porin genes, *ompK35* and *ompK36*, upregulation of *bla*_KPC_, contribute to the development of carbapenems and ceftazidime/avibactam resistance *in vivo*. Overexpression of efflux pumps, acquiring plasmid-carrying *tet (A)* variants, and ribosomal protein change can lead to the adaptive evolution of tigecycline resistance. Specific mutations in chromosomes result in a variety of modifications of LPS, contributing to colistin resistance.

The adaptive evolution of *K. pneumoniae* can be attributed to the impact of the human host’s internal environment and antibiotic selection pressure. The *in vivo* development of antimicrobial resistance in *K. pneumoniae* was established mainly through the acquisition of a resistant plasmid and the emergence of specific mutations. Plasmid-carried resistant genes have been proven to disseminate ubiquitously, such as *bla*_KPC_ and *tet (A)*. Moreover, acquired antimicrobial resistance in hvKP clones demonstrated that *in vivo* adaptive evolution also promotes the convergence of hypervirulence and resistance.

The resistant plasmid might be acquired from the co-infecting or co-colonizing strains, and the internal selection factors may contribute to the horizontal transfer of plasmid within the human host. The emerging resistant *K. pneumoniae* strains *in vivo* could then disseminate through nosocomial settings and be screened by sectional molecular epidemiology studies. It is possible that the internal environment within the human host could serve as an important source of resistant *K. pneumoniae* strains. In the future, more attention should be paid to the *in vivo* genetic process of conversion from antibiotic-susceptible to resistant *K. pneumoniae*, and the high possibility of convergence of hypervirulence and resistance. The evolution of resistant strains could be effectively reduced by blocking this conversion process.

## Author contributions

SL and ML: conceptualization. SL and XF: methodology and writing-original draft preparation. ZS: validation and supervision. SL and ZS: writing-review and editing. ML: project administration. SL: funding acquisition. All authors have read and agreed to the published version of the manuscript.

## Funding

This work was supported by the Ningbo Medical Science and Technology Project, China (Grant no. 2021Y83), the National Natural Science Foundation of China (82272374), and the Ningbo Hangzhou Bay Hospital Qihang Talent Program (Grant no. WY-KY-QH-2021-003).

## Conflict of interest

The authors declare that the research was conducted in the absence of any commercial or financial relationships that could be construed as a potential conflict of interest.

## Publisher’s note

All claims expressed in this article are solely those of the authors and do not necessarily represent those of their affiliated organizations, or those of the publisher, the editors and the reviewers. Any product that may be evaluated in this article, or claim that may be made by its manufacturer, is not guaranteed or endorsed by the publisher.
